# Side-by-side secretion of Late Palaeozoic diverged courtship pheromones in an aquatic salamander

**DOI:** 10.1098/rspb.2014.2960

**Published:** 2015-03-22

**Authors:** Ines Van Bocxlaer, Dag Treer, Margo Maex, Wim Vandebergh, Sunita Janssenswillen, Gwij Stegen, Philippe Kok, Bert Willaert, Severine Matthijs, Erik Martens, Anneleen Mortier, Henri de Greve, Paul Proost, Franky Bossuyt

**Affiliations:** 1 Amphibian Evolution Laboratory, Biology Department, Vrije Universiteit Brussel (VUB), Pleinlaan 2, 1050 Brussels, Belgium; 2 Laboratory of Immunobiology, Department of Microbiology and Immunology, Rega Institute, Katholieke Universiteit Leuven (K.U. Leuven), Minderbroedersstraat 10-Box 1030, 3000 Leuven, Belgium; 3 Laboratory of Molecular Immunology, Department of Microbiology and Immunology, Rega Institute, Katholieke Universiteit Leuven (K.U. Leuven), Minderbroedersstraat 10-Box 1030, 3000 Leuven, Belgium; 4 Structural and Molecular Microbiology, Structural Biology Research Centre, VIB, Pleinlaan 2, 1050 Brussels, Belgium; 5 Structural Biology Brussels, Vrije Universiteit Brussel, Pleinlaan 2, 1050 Brussels, Belgium

**Keywords:** evolution, phylogeny, gene duplications, amphibians, protein pheromones

## Abstract

Males of the advanced salamanders (Salamandroidea) attain internal fertilization without a copulatory organ by depositing a spermatophore on the substrate in the environment, which females subsequently take up with their cloaca. The aquatically reproducing modern Eurasian newts (Salamandridae) have taken this to extremes, because most species do not display close physical contact during courtship, but instead largely rely on females following the male track at spermatophore deposition. Although pheromones have been widely assumed to represent an important aspect of male courtship, molecules able to induce the female following behaviour that is the prelude for successful insemination have not yet been identified. Here, we show that uncleaved sodefrin precursor-like factor (SPF) protein pheromones are sufficient to elicit such behaviour in female palmate newts (*Lissotriton helveticus*). Combined transcriptomic and proteomic evidence shows that males simultaneously tail-fan multiple *ca* 20 kDa glycosylated SPF proteins during courtship. Notably, molecular dating estimates show that the diversification of these proteins already started in the late Palaeozoic, about 300 million years ago. Our study thus not only extends the use of uncleaved SPF proteins outside terrestrially reproducing plethodontid salamanders, but also reveals one of the oldest vertebrate pheromone systems.

## 1. Introduction

**Figure 1.**
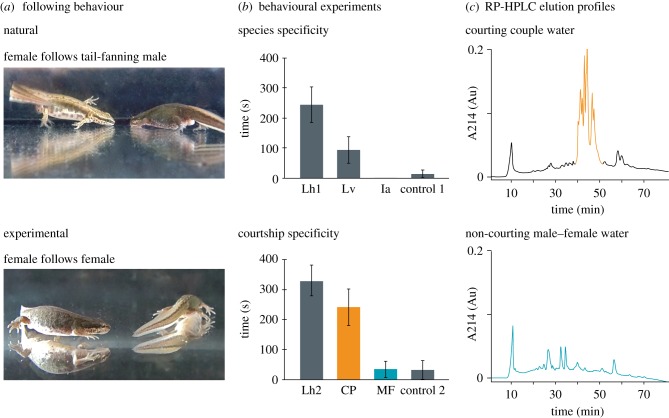
Identification and isolation of *L. helveticus* male courtship pheromones. (*a*) Top: tail-fanning of pheromones towards the nose of the female persuades her to follow the path of the retreating male; bottom: analogous following response of a female in pheromone-containing water during a two-female behavioural bioassay. (*b*) Behavioural assays. Top: two-female tests showing the mean cumulative duration (±s.e.) of *L. helveticus* females' following behaviour in courtship water of their own species (Lh1, *n* = 12), *L. vulgaris* (Lv, *n* = 12) and *I. alpestris* (Ia, *n* = 12), and negative control (control 1, H_2_O, *n* = 11); bottom: two-female tests with *L. helveticus* showing the mean cumulative duration (±s.e.) of *L. helveticus* females' following behaviour in courtship water (Lh2, *n* = 13), RP-HPLC fractions of the courtship-specific peak (CP, orange, *n* = 10), RP-HPLC fractions of a non-courting couple (MF, blue, *n* = 11) and negative control (control 2, H_2_O, *n* = 12). *n* is the number of couples tested. (*c*) Comparison of RP-HPLC profiles of courtship water (top) and male–female (non-courtship) water (bottom). Courtship water shows a courtship-specific peak (orange) that is absent in MF water (blue), indicating that males largely release courtship pheromones (that induce female following) while tail-fanning. Coloured fractions were pooled and used in the two-female courtship specificity tests.

Throughout the animal kingdom, internal fertilization (i.e. the merging of sperm and egg inside the female body) is a widespread reproductive mode that is generally accomplished through copulation, i.e. the insertion of a copulatory organ into the female sex organ [1]. By contrast, males of most advanced salamanders (Salamandroidea, making up about 90% of the more than 650 species of extant salamanders) reproduce by internal fertilization, but deposit a sperm package (spermatophore) on the substrate in the environment, which females subsequently take up with their cloaca. In most families, an enhanced success rate of insemination is accomplished through contact, such as a coordinated tail-straddling walk, or amplexus in which the male sometimes drags the female over the spermatophore [2,3]. However, some male salamanders have abandoned close physical contact altogether and instead largely rely on tail-fanning courtship pheromones to the female [2,4–6]. These pheromones induce following behaviour in the female, which is a prerequisite for the subsequent courtship behaviour (e.g. the male displaying a ‘break’ position, the female touching the male's tail) that culminates in insemination (figure 1*a*; see the electronic supplementary material, movie S1 for the typical behavioural sequence of the courtship process) [7–9].

Although chemical communication is widespread in animal reproduction, female-directed chemical signals that are released when both sexes meet to court are less recognized than attractants, i.e. signals that function to attract partners from a distance [10], or signature mixtures, i.e. signals that are used for recognition of organisms as individuals or members of a particular group [11]. Furthermore, peptide and protein pheromones with various functions (e.g. prompting inter-male aggression, mate guarding, attraction, … see [12,13] for a recent review) have been widely studied across vertebrates and invertebrates, but few studies have demonstrated male proteins that enhance female receptivity [10–13]. In urodeles, the courtship pheromones of only a single family (terrestrial lungless salamanders, Plethodontidae) have been well characterized at the molecular and behavioural level. Males of these species secrete a mixture of two or three distinct protein families (i.e. plethodontid receptivity factor, plethodontid modulating factor, and sodefrin precursor-like factor (SPF)) that have been shown to influence courtship duration by affecting the female's receptivity [14–18]. Within the aquatically reproducing newts (Salamandridae), only a decapeptide with an attractant function in Asian newts of the genus *Cynops* has been intensively studied [19–21]. The decapeptide, termed sodefrin, is derived from a protein precursor that shows sequence similarity with the plethodontid courtship pheromone SPF. However, no studies are available that have characterized pheromones which directly affect female receptivity and induce following behaviour in aquatically reproducing newts. Here, we isolated and purified courtship proteins that are tail-fanned by palmate newts (*Lissotriton helveticus*, Salamandridae) directly from the water, experimentally tested them, and used transcriptomics and phylogenetics to estimate the age of divergence of present-day secreted proteins.

## 2. Material and methods

### (a) Animals

The catching method and the housing conditions were the same as those described elsewhere [9]. Animals were released back to the pond of their origin after the experiments were finished. We used 40 adult males and 40 females of each of the three species of newts (*L. helveticus*, *Lissotriton vulgaris* and *Ichthyosaura alpestris*). All species have an overlapping breeding season and were collected in spring from ponds near Ternat, Belgium. Hybridization and introgression between *L. vulgaris* and *L. helveticus* can occur in sympatric ponds [22], but the occurrence of introgressed individuals in our populations would not essentially affect the outcome of this study.

### (b) Two-female behavioural experiments

We conducted two-female behavioural experiments modified from [9] using *L. helveticus* females. Treer *et al.* showed that two newt females that were kept in water in which a conspecific male had been tail-fanning (henceforth termed courtship water) showed natural courtship responses such as following behaviour and tail touching. With this notion, female courtship responses can be measured in absence of a male, having complete control over the application of candidate pheromones.

We measured following behaviour as the cumulative amount of time in which a female incessantly showed interest towards the other female (including turning towards her, following and tail-touching) during 10 min of observation [9]. This female following behaviour is similar to the courtship response under natural conditions with a courting male, where a female starts to closely follow his movements [7]. Although it cannot be excluded that females not only interact physically, but also use chemical cues [23], this would not influence our results, because such interactions would occur in both the stimulus-containing water and the control.

To evaluate suitability of the animals to be used, all experiments were preceded by a receptivity test before the first experiment (see the electronic supplementary material, receptivity tests). The behavioural experiments with two *L. helveticus* females were done in 800 ml of water, either with or without chemical stimuli. All experiments were performed on consecutive days, at the same time of the day (around 18.00 h), and under the same light (artificial daylight) and temperature (15–18°C) conditions. In all tests, female behaviour was recorded for 12 min, and the first 2 min of the experiment were discarded to allow acclimatization of the animals. Experiments were recorded using a digital camera connected directly to a computer, where the recordings were stored and analysed.

Using different stimuli in the above-mentioned two-female set-up, we conducted sets of behavioural experiments to test: (i) species-specificity, (ii) courtship-specificity, and (iii) functionality of purified SPF.

#### (i) Species specificity

To check whether tail-fanned pheromones are species-specific, we used our two-female test to compare following behaviour of two *L. helveticus* females in courtship water of conspecific males (Lh1) with their behaviour in courtship water of the congeneric species *L. vulgaris* (Lv) and courtship water of the more distantly related *I. alpestris* (Ia; figure 1*b*). Control experiments (control 1) were done in aged tap water that had been kept in the room where the animals were housed, ensuring similar temperature conditions.

Collection of courtship water, containing all molecules that males emit during courtship, was done by putting a receptive male and female in a plastic container (25 × 16 × 14 cm) filled with 800 ml of aged tap water. To obtain similar pheromone concentrations in all experiments, we allowed males to court the females until 20 min of cumulative active tail-fanning was recorded. The couple was then removed and the pheromone-containing courtship water was immediately used in the behavioural experiment.

#### (ii) Courtship specificity

To assess whether female following behaviour is effectively induced by molecules that are released during tail-fanning, we used our two-female test to compare following behaviour of *L. helveticus* females in water containing the reversed-phase high-performance liquid chromatography (RP-HPLC; see section §2d) fractions holding the courtship-specific proteins (CP) versus (i) control water (control 2) and (ii) full courtship water of conspecific males (Lh2), i.e. holding both courtship-specific and non-courtship-specific proteins (figure 1*b,c* and table 1). Finally, we compared (iii) the full range of RP-HPLC fractions of water in which a male and a female had been present for the same amount of time without courtship, containing only non-courtship proteins (MF), with control water (control 2; figure 1*b,c* and table 1).

**Table 1.** Statistical analyses of behavioural experiments. (All behavioural experiments were conducted using two females of *Lissotriton helveticus*. Stimuli are indicated as follows: Lh, *Lissotriton helveticus* courtship water; Lv, *Lissotriton vulgaris* courtship water; Ia, *Ichthyosaura alpestris* courtship water; CP, *Lissotriton helveticus* courtship-specific peak; MF, non-courting male–female water; fraction A, SPF 1 + SPF 3 glycoforms; fraction B, SPF 3 glycoforms only. *n*, the number of couples tested. Statistical analyses (stimulus 1 versus stimulus 2) compare the cumulative duration of observed female following behaviour (in seconds) per couple during 10 min of observation. Bold numbers indicate significance level *p* < 0.01.)

Kruskal–Wallis	stimulus 1	*n*	stimulus 2	*n*	*p* (after Holm–Bonferroni correction)	*u*	*z*
species specificity							
<0.001	Lh1	12	control 1	11	**0**.**002**	19.000	−3.194
	Lh1	12	Lv	12	0.060	41.000	−1.882
	Lh1	12	Ia	12	**0**.**002**	18.000	−3.584
courtship specificity							
<0.001	CP	10	control 2	12	**0**.**003**	12.500	−3.420
	CP	10	Lh2	13	0.528	47.000	−1.117
	MF	11	control 2	12	0.563	60.500	−0.578
functionality of purified SPF							
<0.01	fraction A	10	control 3	11	**0**.**002**	12.000	−3.356
	fraction B	10	control 3	11	**0**.**002**	12.000	−3.356

Protein sampling for these tests was done by placing a male and female in a plastic container (25 × 16 × 14 cm) filled with 600 ml of water. Male–female couples were monitored for courtship behaviour and the amount of time a male fanned his tail was measured. We sampled a minimum of 15 courting couples in which at least 10 min of male tail-fanning occurred. The water of non-courting couples (male–female water, no tail-fanning) was collected in the same way and for the same amount of time as above, but for each animal separately to ensure that there was no courtship display. Subsequent purification of proteins using RP-HPLC is outlined in §2d.

#### (iii) Functionality of purified sodefrin precursor-like factor

**Figure 2.**
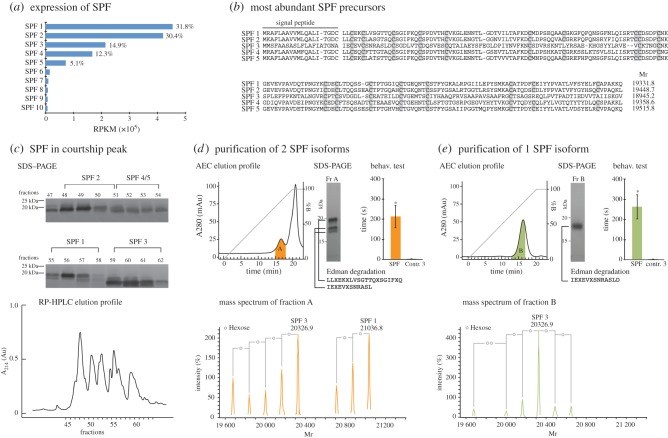
Transcriptomic and proteomic analyses of SPF proteins. (*a*) RNAseq expression level (percentage of total SPF) of the 10 most abundant SPF precursors in the abdominal gland of a male (reads per kilobase per million mapped reads, RPKM). (*b*) MAFFT alignment and theoretical masses of the five most abundant SPF proteins found in the abdominal gland. Cysteins are indicated in grey. (*c*) SDS–PAGE (silver staining) and RP-HPLC elution profile of SPF proteins in a courtship-specific peak. Our analyses show that the SPF proteins present in courtship water match the five most abundant RNA precursors found in the abdominal gland (see the electronic supplementary material, tables S1 and S2). (*d*,*e*) Anion exchange chromatography (AEC) elution profile, silver stained SDS–PAGE, mass spectrometry (deconvoluted mass spectra), Edman sequencing, and behavioural tests of two SPF pheromones (fraction A = SPF 1 + SPF 3 glycoforms) (*d*), and a single SPF pheromone (fraction B = SPF 3 glycoforms only) (*e*). An asterisk indicates significance level *p* < 0.01.

In order to verify that male tail-fanned SPF proteins alone are indeed able to induce female following behaviour, we purified glycoforms of different SPF proteins using RP-HPLC followed by anion exchange chromatography. We tested following behaviour using glycoforms of multiple proteins (fraction A) and glycoforms of a single protein (fraction B; figure 2) versus control water (control 3). Protein sampling for these tests was done as in §2b(ii) and subsequent purification of proteins using RP-HPLC and ion exchange is outlined in §2d.

### (c) Data analysis and statistics

For each of the experiments, we quantified female following behaviour by measuring the cumulative duration that at least one female showed following behaviour during 10 min of observation. The differences in following behaviour were tested with the Kruskal–Wallis test followed by a *post hoc* two-tailed Mann–Whitney *U*-test for pairwise comparisons, and corrected for multiple comparisons using the Holm–Bonferroni method [24]. The analyses were done using IBM SPSS Statistics for Windows (IBM SPSS Statistics for Windows v. 22.0 [25]).

### (d) Purification of proteins

Molecules were extracted by applying non-courtship (male–female) or courtship water onto two separate solid phase extraction cartridges (300 ml per filter; RP-C8 and RP-C18 Sep-Pak plus cartridge, 400 mg sorbent, Waters, Milford, MA) using a vacuum pump. Proteins were eluted from both cartridges with 7.5 ml of 90% (v/v) acetonitrile containing 0.1% (v/v) trifluoroacetic acid (TFA). All acetonitrile was evaporated using a SpeedVac concentrator (SCV-100H, Savant instruments, Farmingdale, NY) for 1 h. The same concentrator was used for all subsequent steps in which acetonitrile was evaporated. After concentration, samples were pooled per condition (courting and non-courting).

For RP-HPLC, pooled and concentrated samples were loaded onto a Source 5RPC column (4.6 × 150 mm, GE Healthcare Life Sciences, Uppsala, Sweden) pre-equilibrated with 0.1% (v/v) TFA (solvent A). After loading, the column was washed for 10 min at a constant flow rate of 1 ml min^−1^ using the same solvent. Proteins were eluted with 80% acetonitrile in 0.1% TFA (solvent B) by applying a linear (from 0% to 100% B in 80 min at 1 ml min^−1^) or flattened gradient (30–65% B in 56 min at 1 ml min^−1^). Detection of eluting proteins was performed at a wavelength of 214 nm, and the eluate was collected in fractions of 1 ml. Fractions of interest were subjected to non-reducing SDS–PAGE using precast gels (Any kD Mini-PROTEAN TGX, Bio-Rad, Hercules, CA). Proteins were visualized by silver staining (Silverquest Silver Staining kit, Invitrogen, Carlsbad, CA). For subsequent behavioural tests with fractions of interest (figure 1*c*, blue and orange), acetonitrile was evaporated.

To further purify the candidate SPF pheromones, RP-HPLC fractions of interest from the courtship water sample were submitted to ion exchange chromatography. After evaporating the acetonitrile, RP-HPLC samples were brought to a pH of 7.5 by addition of buffer containing 20 mM bis–tris propane, 20 mM piperazine and 20 mM *N*-methyl piperazine (Sigma, St. Louis, MO). Samples were loaded onto a 1 ml Hitrap DEAE Fast Flow (GE Healthcare Bio-sciences, Pittsburgh, PA; flow rate 1 ml min^−1^) column pre-equilibrated with binding buffer containing 15 mM bis–tris propane, 15 mM piperazine and 15 mM *N*-methyl piperazine (buffer A, pH 7.5) and washed for at least 10 min with the same buffer until all material in the effluent disappeared. Proteins were eluted with 15 mM bis–tris propane, 15 mM piperazine and 15 mM *N*-methyl piperazine (buffer B, pH 3) by applying a linear gradient (from 0% to 100% B in 20 min). Detection of eluting proteins was performed at a wavelength of 280 nm and the eluate was collected in fractions of 1 ml. Purity of the fractions was assessed by mass spectrometry and non-reducing SDS–PAGE, using precast gels (Any kD Mini-PROTEAN TGX, Bio-Rad). After electrophoretic separation, proteins were visualized by silver staining (Silverquest Silver Staining kit, Invitrogen) and fractions of interest (fractions A and B) were used in two-female tests (figure 2).

### (e) Mass spectrometry and amino acid sequence analyses

Mass analyses of the HPLC fractions were performed by electrospray ionization mass spectrometry on an ESQUIRE- LC MS ion trap (Bruker, Brussels, Belgium). In addition, mass analyses of the desalted ion exchange fractions (Zip Tip C18, 10 μl, Millipore, Bedford, MA) were performed on an Amazon Speed electron transfer dissociation ion trap mass spectrometer. Characterization of the glycan moiety was done through in-source fragmentation on the Esquire ion trap mass spectrometer by gradually elevating the potential on skimmer 1 and the exit caps in the electrospray source. Averaged profile spectra of proteins were deconvoluted with the Bruker software Data Analysis v. 4.1. To identify the level of glycosylation, the experimentally determined average relative molecular mass (Mr) of the SPF proteins was compared with the theoretical average Mr of the SPF cDNA precursors found in the abdominal gland.

Peak fractions of courtship water were subjected to a non-reducing SDS-PAGE using precast gels (any kD Mini-PROTEAN TGX, Bio-Rad). After electrophoresis, proteins were transferred from the gel onto a polyvinylidene difluoride membrane by semi-dry blotting (Trans Blot Turbo System, Bio-Rad) and stained with 0.1% Coomassie brilliant blue R-250 (Sigma; membrane not shown). All protein bands were excised from the blot for N-terminal amino acid sequencing (Edman sequencing) on a 491 Procise cLC protein sequencer (Applied Biosystems, Foster City, CA).

### (f) Transcriptomics and gene expression estimates

The pheromone-producing, sexually dimorphic abdominal glands from a single male were sampled for RNA sequencing (RNA-seq). Total RNA was extracted using TRI Reagent (Sigma-Aldrich) and the RNAeasy mini kit (Qiagen). A pair-end PE50 cDNA sequencing library was created (Baseclear, Leiden, The Netherlands) with Illumina TruSeq RNA library preparation kit and 52 040 842 fragments were sequenced on an Illumina HiSeq 2500 instrument. FastQ reads were generated after analyses with Illumina Casava pipeline (v. 1.8.3), a post-filtering script and FASTQC quality control tool (v. 0.10.0) to remove low quality, PhiX-control and adapter reads. De novo transcriptome assembly was performed with Trinity [26]. SPF sequences were identified by BLASTing assembled transcripts against an in-house database, and through aligning them to a dataset containing SPF sequences from the Uniprot database using RAPsearch [27].

Rapid amplification of cDNA ends (RACE) was performed to obtain complete protein sequences from different SPF precursors. Primers were designed on the 3′-untranslated region to amplify full-coding sequences of SPF transcripts as follows:

SPF_primer_A, 5′-TTGTTAATAAWYATTCTGTAAAGARGCT-3′;

SPF_primer_B, 5′-GCCTTGTTGBCAAAAHKTCTTC-3′;

SPF_primer_C, 5′-ACAAYTWCTAAGCTGGHKTAGGA-3′;

SPF_primer_D, 5′-GTGTGTATWTGRGGTATRAACAAAGGTC-3′,

SPF_primer_E, 5′-CCAACAATTACTRRGMKGGAGTAGG-3′;

SPF_primer_F, 5′-CAACTACTAAGCTRRAGTMRGAGTGC-3′;

SPF_primer_G, 5′-GGRTAGGATTGCGTCAGATGTT-3′;

SPF_primer_H, 5′-TAGGAATGTTTCTAYKGACKACTACTRAG-3′;

SPF_primer_I, 5′-CTATTGCTAAGCTGKGGTG-3′;

SPF_primer_J, 5′-GCTGGCACATGGGCATGT-3′;

SPF_primer_K, 5′-GCCCAWACASKACTAAGCACATT-3′;

SPF_primer_L, 5′-GACTCTGVATTHCAGGTACTTGTAGAG-3′.

One microgram total RNA from the same extraction procedure as in RNA-seq was used to create RACE cDNA with the SMARTer-RACE cDNA amplification kit (Clontech). PCR products were amplified with FastStart high fidelity Taq DNA polymerase (Roche). Amplification products were cloned into a pGEM-T Easy cloning vector (Promega), and vectors were transformed into TOP10 Competent Cells (Invitrogen). Colonies were picked randomly and inserts were amplified with FastStart Taq DNA polymerase. Amplification products were purified and sequenced by the VIB genetic service facility (Antwerp, Belgium). For comparison with protein masses found in courtship water, contiguous sequences (contigs) were assembled with CodonCode Aligner v. 3.7.1.1 (CodonCode Corporation) using a 99% similarity threshold, after quality trimming. Signal peptides, predicted using SignalP 4.0 [28], were removed and protein masses were calculated with the *p*I/*M*_w_ tool on Expasy (http://web.expasy.org/compute_pi/). Finally, levels of SPF gene expression were estimated using RNA-seq read counts on the SPF transcripts obtained from our RACE procedure. Expression levels were determined using reads per kilobase per million mapped (RPKM) reads values [29].

### (g) Phylogeny and divergence time estimates

We combined protein sequences of a representative set of 16 SPF precursors of *L. helveticus* found in this study with available sequences (representing the major currently available evolutionary lineages) of SPF precursors of four plethodontid (GenBank nos. AAZ06338, AAZ06329, AAZ06335, AAZ06331), three ambystomatid (GenBank nos. CN040015, CN041146, CN048649) and six other *Lissotriton* sequences available on GenBank (i.e. two *Lissotriton montandoni*, GenBank nos. ACB54670 and ACB54672, and four *L. vulgaris*, GenBank nos. ACB54665, ACB54666, ACB54668 and ACB54669). Two frog sequences (GenBank nos XP_002943341, F6PQG9) were chosen as outgroup. Alignment of the protein sequences was done with MAFFT [30] using the L-INS-i method (automatically assigned) and resulted in a data matrix of 216 characters. Maximum-likelihood (ML) analyses were run in PAUP* [31], using an LG amino acid rate matrix, with empirical frequencies, estimated proportion of invariable sites (0.0527345) and distribution of rates at variable sites following a gamma distribution with four categories and estimated shape parameter (1.79548). This resulted in a single ML tree with likelihood score −ln *L* = 8271.589. Bayesian analyses and Bayesian posterior probabilities were calculated in MrBayes [32]. Two runs of four Markov chain Monte Carlo chains each were executed in parallel for 5 000 000 generations, with a sampling interval of 500 generations and a burn-in corresponding to the first 1 000 000 generations. Convergence of the parallel runs was confirmed by split frequency standard deviations (less than 0.01) and potential scale reduction factors (approximating 1.0) for all model parameters. Adequate posterior sampling was verified using Tracer v. 1.5 [33], by checking if the runs had reached effective sampling sizes of more than 200 for all model parameters. Speciation-duplication analyses were done using Notung [34]. To estimate the age of the earliest diversification in our SPF pheromones, we used a Bayesian-relaxed molecular clock model (uncorrelated lognormal) implemented in BEAST [35] that accounts for lineage-specific rate heterogeneity. As a calibration point, we used the divergence of the (Salamandridae, Ambystomatidae) clade from Plethodontidae, a relationship that is widely accepted [36–40] and was also recovered in our ML tree. We used the mean (175.7 Ma) and standard deviation (14.8 Ma) of the last five studies [36–40] presented on Timetree [41] (version of 13 January 2014) to calibrate this node in our tree with a central 95% range of 146.7–204.7 Ma (calculated in BEAST). Posterior sampling was verified using Tracer v. 1.5 [33]. The sodefrin precursor sequence of *Cynops* was not included, because the end of the sequence (containing the sodefrin peptide) is not homologous with our full-length proteins.

## 3. Results and discussion

### (a) Salamandrid courtship pheromones progressively evolve towards species-specificity

We first used our two-female behavioural test to assess whether water in which a male had been tail-fanning (courtship water) was able to induce following behaviour in conspecific females. The set-up of this assay removes the secondary sexual morphological and chemical characteristics, as well as the visual cues of tail-fanning of a male, while retaining the required presence of another individual necessary to exhibit following behaviour. Our behavioural assays with two palmate newt females showed that courtship water of this species induced significantly more following behaviour than control water (figure 1*b* and table 1; Lh1, *n* = 12; control 1, *n* = 11; *p* < 0.01). These tests confirm that courtship water is able to induce female courtship responses in palmate newts, even in the absence of the male secondary sexual characteristics and visual cues associated with tail-fanning. Next, we checked whether tail-fanned pheromones are species-specific by measuring following behaviour of *L. helveticus* females in courtship water of *L. vulgaris* and *I. alpestris.* Females of palmate newts show a reduced response in courtship water of the congeneric species *L. vulgaris* (figure 1*b* and table 1; Lh1, *n* = 12; Lv, *n* = 12; *p* = 0.060). Hybridization and introgression between both species can occur in sympatric ponds [22] and may partially explain these observations. However, female palmate newts showed no following behaviour in courtship water of the more distantly related alpine newts (*I. alpestris*; figure 1*b* and table 1; Lh1, *n* = 12; Ia, *n* = 12; *p* < 0.01, suggesting that tail-fanned courtship pheromones progressively evolve towards species-specificity.

### (b) Uncleaved glycosylated sodefrin precursor-like factor proteins induce female courtship behaviour in palmate newts

We sampled proteins emitted during male tail-fanning directly from water and performed RP-HPLC to compare elution profiles of courtship water with that of water in which a non-courting male and female had been held. These analyses show a recurrent pattern of elution profiles showing a peak that is present in water in which a male has been tail-fanning (more than 20 profiles, one shown; figure 1*c*, courting couple, courtship-specific peak in orange), but absent in water in which non-courting males and females were held for the same amount of time (five profiles, one shown; figure 1*c*, non-courting male–female, blue). We also sampled courtship water at the end of the breeding season and observed a peak with a much lower absorbance, indicating a decrease in tail-fanned proteins towards the end of the breeding season (see the electronic supplementary material, figure S1). A behavioural assay with the RP-HPLC fractions of the courtship-specific peak (CP, figure 1*c*, in orange) indicated that they induced female following behaviour significantly more than control water (figure 1*b* and table 1; CP, *n* = 10; control 2, *n* = 12; *p* < 0.01), but not significantly different from full courtship water (figure 1*b* and table 1; CP, *n* = 10; Lh2, *n* = 13; *p* = 0.528). Conversely, RP-HPLC fractions of non-courting male–female water (MF, figure 1*c*, blue) resulted in female reactions that were not significantly different from those in control water (figure 1*b* and table 1; MF, *n* = 11; control 2, *n* = 12; *p* = 0.563). These observations indicate that the RP-HPLC fractions of the courtship-specific peak effectively contain the male courtship pheromones that can induce female following behaviour. N-terminal amino acid sequencing (Edman sequencing) of these pooled fractions indicated the presence of multiple isoforms of the SPF family (see the electronic supplementary material, table S1). These proteins were considered ideal pheromone candidates, because a full-length protein (i.e. not cleaved, except for the signal peptide) of this family identified from the mental gland of the terrestrially reproducing plethodontid salamander *Desmognathus ocoee* was shown to increase female receptivity during courtship [15].

We further characterized the diversity of SPF proteins by combining transcriptome analyses of the pheromone-producing, sexually dimorphic abdominal gland of a single male with proteome analyses of RP-HPLC fractions of the courtship-specific peak. Whole transcriptome sequencing (RNAseq) of this male gland and de novo assembly of nearly 52 million (Mio) reads revealed 4.1 Mio reads (7.9%) belonging to this SPF family of molecules. RACE-PCR sequencing revealed 32 different cDNA precursor sequences (GenBank numbers KJ402326–KJ402357) encoding for 31 unique mature proteins. RNAseq expression analyses indicated five isoforms as most abundant, together making up 94.5% of the SPF transcripts identified in the transcriptome (figure 2*a*,*b*). Interestingly, the pairwise amino acid divergences between these sequences were between 19.2% and 78.8%, indicating that these proteins do not only result from allelic variation.

To determine the presence of post-translational modifications and to confirm that these precursors are also effectively translated and tail-fanned to the female, we performed an RP-HPLC with a prolonged gradient (figure 2*c*), which gave a better separation of the SPF proteins, and combined mass spectrometry analyses with Edman sequencing of individual fractions. Mass spectrometry analyses indicated the presence of an oligosaccharide with 2 *N*-acetylglucosamine units (GlcNAc) and multiple hexoses (see the electronic supplementary material, figure S2) attached to the available glycosylation sites of the proteins. Individual RP-HPLC fractions revealed the presence of multiple proteins for which the glycosylated masses (up to eight hexoses) match the theoretically predicted masses derived from the five most abundant cDNA precursors (see the electronic supplementary material, table S2). Additionally, several of these predictions could be confirmed by N- terminal amino acid sequencing (see the electronic supplementary material, table S1). This indicates that SPF is effectively present as multiple uncleaved (i.e. except for the signal peptide) proteins with different levels of glycosylation (glycoforms) in the courtship-specific peak.

Next, we performed ion exchange chromatography to purify SPF from the courtship-specific peak fractions. SDS–PAGE, mass spectrometry and Edman sequencing all indicated that this led to removal of non-SPF as well as some of the SPF proteins, and resulted in a sample containing two SPF proteins (SPF 1: Mr = 21036.8; SPF 3: Mr = 20326.9) with multiple glycoforms (figure 2*d*). A two-female behavioural experiment with these proteins resulted in a significant increase of female following behaviour compared to control water (figure 2*d* and table 1; fraction A, *n* = 10; control 3, *n* = 11; *p* < 0.01), and confirms that SPF proteins alone are able to induce female following, even in the absence of visual stimuli of a courting male. Finally, we used the same techniques to purify a single SPF isoform (SPF 3: Mr = 20326.9) with its glycoforms (figure 2*e*). A two-female behavioural experiment with this protein induced female following behaviour significantly more than control water (figure 2*e* and table 1; fraction B, *n* = 10; control 3, *n* = 11; *p* < 0.01). This experiment indicates that glycoforms of a single SPF protein are able to elicit female courtship responses. Future investigations on the relative ability of individual SPF proteins and glycoforms to induce female following in the palmate newt and related species could give important insights in the evolution of species-specificity of protein pheromones.

### (c) Side-by-side secreted sodefrin precursor-like factor-proteins originated from a Late Palaeozoic gene duplication

**Figure 3.**
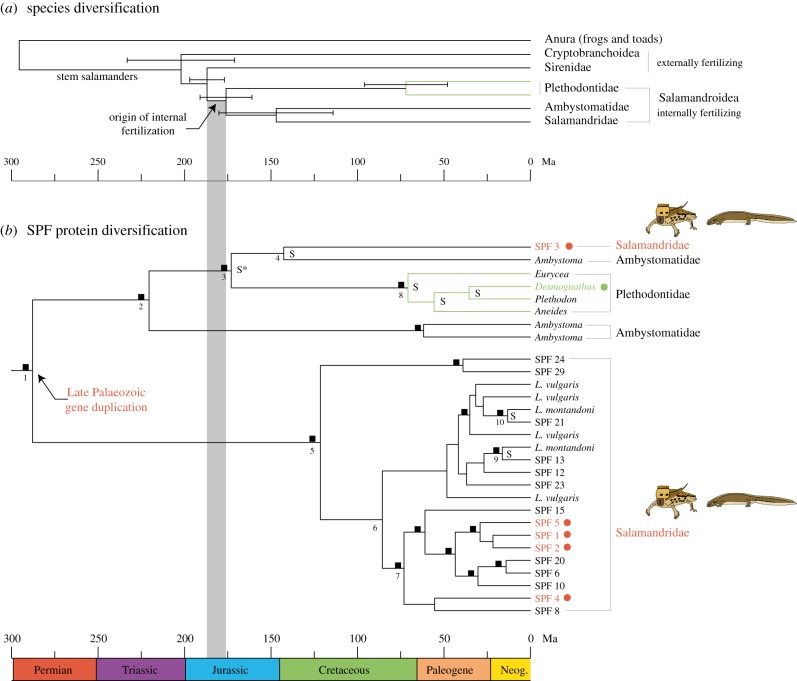
Time estimates. (*a*) Species diversification. The mean and standard deviations for species diversifications were calculated from the last five published estimates [36–40]. The origin of internal fertilization in the ancestor of Salamandroidea is indicated in grey. (*b*) SPF protein diversification. The tree shows Bayesian dating estimates, the asterisk denotes the calibration point. The plethodontid precursors were chosen to reflect the largest known SPF diversity in this family. The fact that our gene tree of plethodontid SPFs corresponds to the higher taxonomic level relationships of these species therefore indicates that the known SPF variation is the result of family-specific variation and/or gene duplications (in agreement with Palmer *et al*. [14]). Bayesian posterior probabilities >95 are indicated with black squares. Speciation nodes are indicated with S, all other nodes are considered duplication nodes (by Notung analyses). Age estimates of numbered nodes are presented in the electronic supplementary material, table S3. Numbered SPFs all indicate sequences from the abdominal gland of the palmate newt *L. helveticus*. The top five expressed proteins, which were also confirmed in courtship water, are indicated with red circles. The green circle denotes the species in which the pheromone function was demonstrated in plethodontids.

Phylogenetic analyses combining our palmate newt SPF cDNA precursors with the available sequences on GenBank confirm that SPF diversification goes beyond allelic variation by indicating multiple gene duplication events (figure 3). Speciation-duplication analyses identified speciation events that conform to established higher-level phylogenetic relationships of salamanders (figure 3*a*), but also recognized two duplications (figure 3*b*, nodes 1 and 2) that occurred before the Plethodontidae–Salamandridae divergence (figure 3*b*, node 3). The strongly supported relationship of SPF proteins from lungless salamanders (Plethodontidae) with a clade uniting an *Ambystoma* and our salamandrid SPF3 precursors (figure 3*b*, indicated with an asterisk) reveals orthologues corresponding to the Salamandridae–Plethodontidae divergence [36–41], and defines a split (figure 3*b*, node 3) that had remained unidentified in previous SPF studies. We used the mean and standard deviation (175.7 ±14.8 Ma) of the last five studies presented on Timetree (version of 13 January 2014) to calibrate this node with a central 95% range of 146.7–204.7 Ma, and estimated precursor divergence times with a Bayesian-relaxed molecular clock model implemented in BEAST [35]. Our results reveal a Late Palaeozoic gene duplication event that denotes the early onset of SPF diversification and secretion (the latter as indicated by our protein characterization from courtship water, figure 3*b* red circles) at about 288.4 Ma (95% HPD = 200.6–385.1 Ma; figure 3*b*, node 1). Our time estimates for salamander speciation nodes in the gene tree (see the electronic supplementary material, table S3) are close to the mean of the last five studies [36–40] in Timetree [41] that estimated the divergence times for both the Ambystomatidae–Salamandridae split (this study, node 4: 143.4 Ma; Timetree: 146.8 Ma) and the onset of diversification of Plethodontidae (this study, node 8: 70.6 Ma; Timetree: 72.1 Ma). Additionally, the two nodes that represent the same speciation event in *Lissotriton* in our gene tree (figure 3*b*, nodes 9 and 10, *L. vulgaris* versus *L. montandoni*) have similar age estimates (16.3 and 13.2 Ma, respectively). All these results together strengthen confidence in our divergence time estimates, including the duplication events of SPF genes. Our timing of the earliest SPF divergence therefore is close to the origin of stem salamanders (Anura–Caudata divergence, figure 3*a*, estimated at 295.5 ± 21 Ma from the mean of the last five studies [37,39,40,42,43] in Timetree [41]) and considerably predates the currently known use of this protein system (i.e. in crowngroup plethodontids, figure 3, green circle and branches).

Our study not only characterizes the pheromones behind the intriguing female following behaviour in salamandrid newts, but also expands the evidence for the use of uncleaved SPF pheromones from a single family (Plethodontidae) to potentially all salamanders. Uncleaved SPF proteins until now were shown to be functional as a pheromone in a single plethodontid species *D. ocoee* (figure 3*b*, green circle) [15]. Although an SPF-derived pheromone was initially discovered in a salamandrid, the cleaved active decapeptide (sodefrin, an attractant in *Cynops*) originated through a translational frame shift and as a consequence shows no homology with uncleaved SPF protein pheromones. Additionally, the short peptide obtained its pheromone function in the genus *Cynops*, and therefore independently from uncleaved proteins [44]. To the best of our knowledge, our study of an aquatic salamandrid is the first to expand the effective behavioural evidence for a courtship pheromone function of uncleaved SPF proteins outside the family of plethodontids. Additionally, the side-by-side secretion of anciently diverged proteins (figure 3*b*, red circles) in our newt species suggests that the courtship function for these proteins considerably predates the Salamandridae–Plethodontidae divergence. Although cDNA studies in individual species already indicated the presence of multiple isoforms, the known diversity of SPF precursors in each of these families resulted from family-specific gene duplications and/or polymorphisms [14,45]. By contrast, our palmate newts tail-fan proteins (figure 3*b*, red circles) of which the estimated divergence dates back to the Late Palaeozoic (figure 3*b*, node 1) and our results therefore suggest a pheromone function for these molecules already in the earliest salamanders, about 300 Ma.

Our combined evidence indicates that, although very different courtship behaviours can be observed across the evolutionary tree of salamanders [3,6,15,19], the function of uncleaved SPF proteins to regulate female sexual receptivity originated early in salamander evolution and has been conserved ever since. The constant presence of multiple copies of SPF shows that species-specificity of this pheromone system may have been maintained through amino acid sequence evolution in individual proteins, variation in the proportion of these proteins or a combination of both, potentially further fine-tuned by variation in glycosylation patterns.

## Supplementary Material

Electronic Supplementary Material
