# Supplementary material for: Side-by-side secretion of Late Palaeozoic diverged courtship pheromones in an aquatic salamander
Source: Proc Biol Sci. 2015 Mar 22;282(1803):20142960. doi: 10.1098/rspb.2014.2960 (PMC4345460; doi:10.1098/rspb.2014.2960)
Supplement: Electronic Supplementary Material [file rspb20142960supp1.pdf]

Electronic Supplementary Material

Side-by-side secretion of late Palaeozoic diverged courtship pheromones in an aquatic salamander

Ines Van Bocxlaer[1*], Dag Treer[1*], Margo Maex[1*], Wim Vandebergh[1], Sunita Janssenswillen[1], Gwij Stegen[1], Philippe Kok[1], Bert Willaert[1], Severine Matthijs[1], Erik Martens[2], Anneleen Mortier[3], Henri de Greve[4,5], Paul Proost[3] and Franky Bossuyt[1]

1 Amphibian Evolution Lab, Biology Department, Vrije Universiteit Brussel (VUB), Pleinlaan 2, B-1050 Brussels, Belgium

2 Laboratory of Immunobiology, Department of Microbiology and Immunology,
Rega Institute, Katholieke Universiteit Leuven (K.U. Leuven), Minderbroedersstraat 10 - box 1030, B-3000 Leuven, Belgium

3 Laboratory of Molecular Immunology, Department of Microbiology and Immunology,
Rega Institute, Katholieke Universiteit Leuven (K.U. Leuven), Minderbroedersstraat 10 - box 1030, B-3000 Leuven, Belgium

4 Structural and Molecular Microbiology, VIB Department of Structural Biology, VIB, Pleinlaan 2, 1050 Brussels, Belgium.

5 Structural Biology Brussels, Vrije Universiteit Brussel, Pleinlaan 2, 1050 Brussels, Belgium

*** These authors contributed equally to this work**

Keywords: evolution, phylogeny, gene duplications, amphibians, protein pheromones

Author for Correspondence: Franky Bossuyt; e-mail: fbossuyt@vub.ac.be

1. Behaviour and experiments

Behaviour

Natural and experimental behaviour in palmate newts is described in the main paper, and can be seen in **Movie S1**:
http://www.amphibia.be/downloads/abcde/pheromones.mov

Receptivity tests

To evaluate suitability of the animals to be used, all experiments were preceded by a receptivity test before the first experiment. Receptivity was tested by putting a male and a female together in a plastic container (25×16×14 cm) filled with 800 ml of aged tap water. Males that induced female following were considered receptive, and were used for collection of courtship water and chemical stimuli. Only females that followed a courting male were selected for subsequent (positive and control) two-female tests. For the species-specificity experiments, the courtship water of *L. vulgaris* and *I. alpestris* (used to test *L. helveticus* females) was first tested in a two-female test with *L. vulgaris* and *I. alpestris*, respectively. The positive reaction of these species to their own courtship water assured that the reduced reaction of *L. helveticus* females was due to species-specificity only, and not due to experimental variation in the potency of courtship water.

2. SPF expression

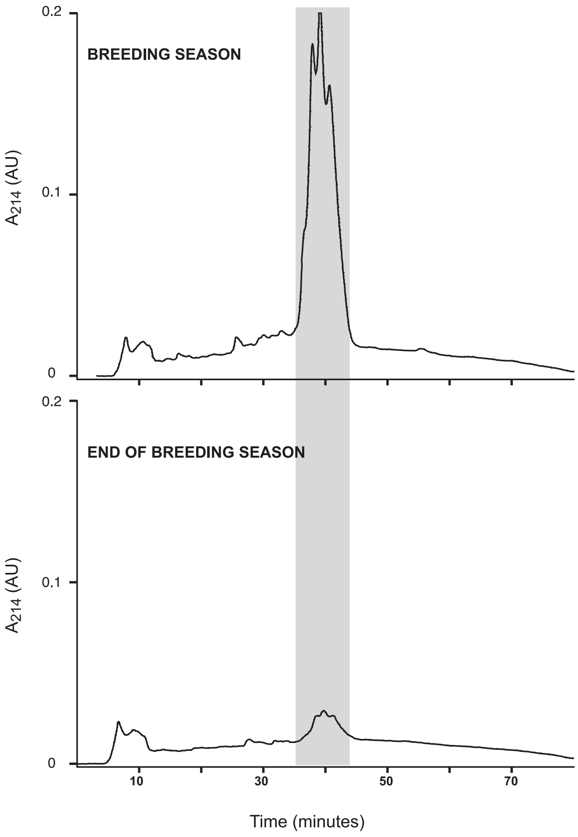

Figure S1. SPF expression in courtship water during and at the end of the breeding season. We performed RP-HPLC to purify SPF proteins from courtship water and compared their abundance in courtship water taken during the highlight of the breeding season with that sampled towards the end of the breeding season (when males are still tail-fanning). Comparison of RP-HPLC elution profiles indicates that the SPF content is strongly reduced at the end of the breeding season (grey bar). Given that we compare molecules from courtship water, i.e. molecules that were effectively tail-fanned by the male to the female, this observation strengthens the evidence for a courtship function of SPF proteins during the breeding season.

3. Identification of SPF proteins and post-translational modifications

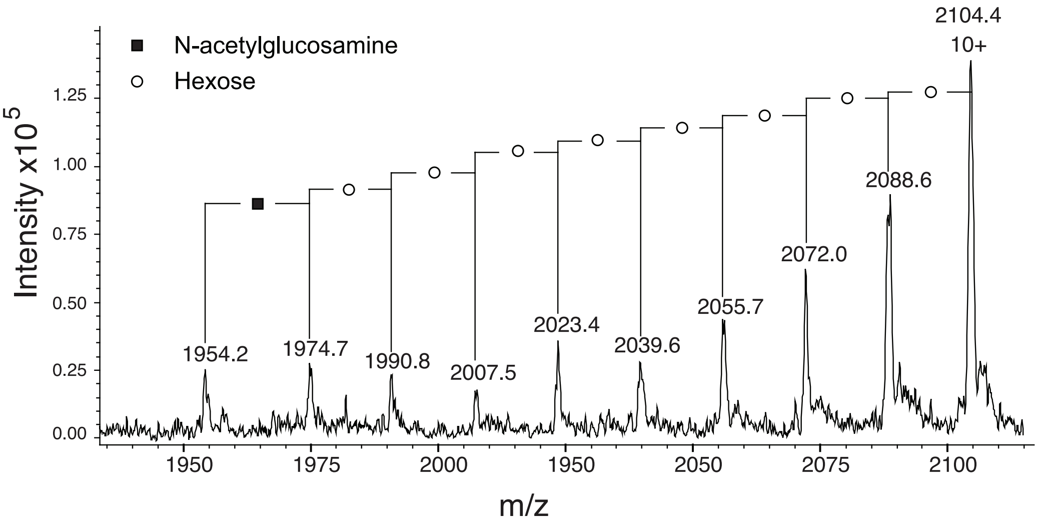

Figure S2. In source fragmentation pattern of SPF used to determine post-translational modifications. Eight hexoses (Mr of one hexose: 162.14) and one core N-acetylglucosamine (GlcNAc; Mr: 203.19) could be fragmented from a ten-times charged ion (m/z 2104.4). Taking into account the eight hexoses and two GlcNAcs, the m/z value of 2104.4 corresponds to the ion with 10 charges of SPF 1 ([[2104.4*10]-10]-[8*162.14]-[2*203.19]] = 19330.5). Typically, N-linked glycans contain two GlcNAcs. However, the glycosidic bond between the second core GlcNAc and the protein moiety rarely breaks during in-source fragmentation, thus no signal corresponding to the removal of the second core GlcNAc was detected. The N-linked glycan structure is most likely of the high mannose type, more specifically a Man(9 or 8)GlcNAc(2)-glycan.

Table S1. Edman sequences. Cysteines were not detected (indicated as X in the sequence) as they were not alkylated prior to the sequence analysis.

SPF in courtship peak (cf. Fig. 2C)	
SPF1	LLXEKXLVSGTTQXSGIFKQX
SPF2	LLXETXLASGTSQXS
SPF2	LLXETXLA
SPF3	IEXEVXSNRASLDXSGDLV
SPF5	ILXEKXLATSTTQXSXIFKQ
SPF5-like	ILSEK(T)FATSTTQ
SPF20	LLxEKxLASGTTQXS
SPF20	LLxEKxLASGTTQXSGIFKQ
Purified SPF (cf. Fig. 2D)	
SPF1	LLXEKXLVSGTTQXSGIFXQ
SPF3	IEXEVXSNRASL
SPF3	IEXEVXX
Purified SPF (cf. Fig. 2E)	
SPF3	IEXEVXSNRASLD

Table S2. Protein masses and identification of the level of glycosylation

Full analysis of mass spectrometry data of SPF proteins in the courtship specific peak (fraction numbers refer to figure 2c), with corresponding cDNA precursor sequence matches from the abdominal gland. The table states (i) the measured relative molecular masses (Mr), (ii) the calculated precursor masses taking into account disulfide bridges and removal of the signal peptide, and (iii) the calculated precursor masses with glycosylation, taking into account S-S bridges and removal of the signal peptide. None of the precursor amino acid (AA) sequences shows homology with the 10 AA fragment that codes for the attractant deca-peptide sodefrin in *Cynops pyrrhogaster*, indicating that palmate newts do not express such a peptide.

	SPF found in courtship water (CP)	Match with precursor found in abdominal gland				
**Fraction**	**i. Measured Mr**	**Precursor**	**ii. Calculated Mr precursor**	**GlcNAc (Mr 203.19)**	**Hexose (Mr 162.1442)**	**iii. Calculated Mr glycosylated precursor**
47	20681.8	No match A				
47	20843.7	No match A + hex				
48	20830.0	SPF 2	19448.7	2	6	20828.0
48	20667.3	SPF 2	19448.7	2	5	20665.8
49	20830.4	SPF 2	19448.7	2	6	20828.0
49	20667.5	SPF 2	19448.7	2	5	20665.8
50	20829.7	SPF 2	19448.7	2	6	20828.0
50	20667.6	SPF 2	19448.7	2	5	20665.8
51	20697.3	No match B				
51	20828.4	SPF 2	19448.7	2	6	20828.0
51	20738.7	SPF 4	19358.6	2	6	20737.9
51	20667.0	SPF 2	19448.7	2	5	20665.8
52	20696.8	No match B				
52	20737.4	SPF 4	19358.6	2	6	20737.9
52	20896.5	SPF 5	19515.8	2	6	20895.0
52	21059.7	SPF 5	19515.8	2	7	21057.2
52	21028.2	No match C				
53	20897.6	SPF 5	19515.8	2	6	20895.0
53	21059.0	SPF 5	19515.8	2	7	21057.2
53	20865.8	No match C- hex				
53	20834.7	No match D				
53	21028.2	No match C				
53	20797.5	No match E				
53	20698.8	No match B				
54	20985.7	No match F				
54	20896.1	SPF 5	19515.8	2	6	20895.0
54	20868.2	No match C- hex				
54	20834.9	No match D				
55	21037.7	SPF 1	19331.8	2	8	21035.3
55	20981.2	No match F				
55	20874.2	SPF 1	19331.8	2	7	20873.2
55	20713.6	SPF 1	19331.8	2	6	20711.1
56	21037.6	SPF 1	19331.8	2	8	21035.4
56	20875.5	SPF 1	19331.8	2	7	20873.2
56	20713.1	SPF 1	19331.8	2	6	20711.1
57	20876.3	SPF 1	19331.8	2	7	20873.2
57	20924.7	No match G				
57	21038.4	SPF 1	19331.8	2	8	21035.4
57	20712.8	SPF 1	19331.8	2	6	20711.1
58	20876.1	SPF 1	19331.8	2	7	20873.2
58	20924.2	No match G				
58	21035.2	SPF 1	19331.8	2	8	21035.4
58	20712.3	SPF 1	19331.8	2	6	20711.1
59	20327.1	SPF 3	18945.2	2	6	20324.4
59	20164.5	SPF 3	18945.2	2	5	20162.3
60	20326.5	SPF 3	18945.2	2	6	20324.4
60	20163.7	SPF 3	18945.2	2	5	20162.3
60	20003.4	SPF 3	18945.2	2	4	20000.1
60	19679.1	SPF 3	18945.2	2	2	19675.8

4. Molecular dating estimates

Table S3. **Age estimates and 95% highest posterior density (HPD).** Node numbers correspond to the time tree of SPF protein diversification in figure 3.

	Node	Mean	95% HPD
Duplication 1	1	288.4	200.6 - 385.1
Duplication 2	2	220.8	165.2 - 282.4
Plethodontidae *vs.* (Ambystomatidae, Salamandridae)	3	173.0	145.0 - 204.0
Ambystomatidae *vs.* Salamandridae	4	143.4	98.4 - 186.8
Duplication 3	5	121.9	71.7 - 175.5
Duplication 4	6	85.9	54.0 - 122.8
Duplication 5	7	73.3	45.3 - 103.1
Crowngroup Plethodontidae	8	70.6	41.7 - 101.2
L. vulgaris vs *L. montandoni*	9	16.3	7.2 - 27.0
L. vulgaris vs *L. montandoni*	10	13.2	6.1 - 21.2
